# Drift Removal for Improving the Accuracy of Gait Parameters Using Wearable Sensor Systems

**DOI:** 10.3390/s141223230

**Published:** 2014-12-05

**Authors:** Ryo Takeda, Giulia Lisco, Tadashi Fujisawa, Laura Gastaldi, Harukazu Tohyama, Shigeru Tadano

**Affiliations:** 1 Division of Human Mechanical Systems and Design, Faculty of Engineering, Hokkaido University, Sapporo 060-8628, Japan; E-Mail: r.takeda@eng.hokudai.ac.jp; 2 Department of Mechanical and Aerospace Engineering, Politecnico di Torino, Torino 10129, Italy; E-Mails: giulia.lisco@polito.it (G.L.); laura.gastaldi@polito.it (L.G.); 3 Division of Human Mechanical Systems and Design, Graduate School of Engineering, Hokkaido University, Sapporo 060-8628, Japan; E-Mail: tadashi-fujisawa@ec.hokudai.ac.jp; 4 Department of Health Science, Hokkaido University School of Medicine, Sapporo 060-0812, Japan; E-Mail: tohyama@med.hokudai.ac.jp

**Keywords:** gait analysis, biomechanics, wearable sensors, drift effect, Lower Limb Kinematics, spatio-temporal gait parameter

## Abstract

Accumulated signal noise will cause the integrated values to drift from the true value when measuring orientation angles of wearable sensors. This work proposes a novel method to reduce the effect of this drift to accurately measure human gait using wearable sensors. Firstly, an infinite impulse response (IIR) digital 4th order Butterworth filter was implemented to remove the noise from the raw gyro sensor data. Secondly, the mode value of the static state gyro sensor data was subtracted from the measured data to remove offset values. Thirdly, a robust double derivative and integration method was introduced to remove any remaining drift error from the data. Lastly, sensor attachment errors were minimized by establishing the gravitational acceleration vector from the acceleration data at standing upright and sitting posture. These improvements proposed allowed for removing the drift effect, and showed an average of 2.1°, 33.3°, 15.6° difference for the hip knee and ankle joint flexion/extension angle, when compared to without implementation. Kinematic and spatio-temporal gait parameters were also calculated from the heel-contact and toe-off timing of the foot. The data provided in this work showed potential of using wearable sensors in clinical evaluation of patients with gait-related diseases.

## 1. Introduction

Gait analysis is a widely used clinical tool for quantifying human walking ability, by identifying differences in gait patterns and parameters. Currently the most popular method of gait analysis is done with reflective skin markers and camera based motion analysis system such as Vicon (Vicon Motion Systems, Inc., Los Angeles, CA, USA). The combined use of stereo photogrammetry and reflective skin markers allows to calculate the joint kinematics and to assess the markers trajectories in time. However, the use of optical tracking systems has been hindered in practice by appropriate laboratory techniques, by the time necessary for the measurements, by the markers' occlusion and the prohibitive costs. Furthermore, the worst drawback regards the subject's constrained freedom of movement, where the subject is required to move inside a restrained space and in a controlled environment [1].

An alternative method for gait analysis is to use wearable sensor systems [2–5]. This method has the advantage of identifying human motion virtually anywhere, both indoors and outdoors. For instance, one of the outdoor applications made possible with the use of inertial sensors concerns the sport sciences field, such as during in-field motion capture measurements that do not allow the use of a markers-based motion analysis, as reported by Gastaldi *et al.* [6]. However, these methods do not directly measure position, but only kinetic data (acceleration, angular velocity, magnetic fields, *etc.*) of body segments they are attached to. Therefore, translating the collected data into meaningful kinematic ones that can be used for diagnosing patients has been the challenge in the field of biomechanics. A sensor to body calibration procedure is needed in order to study joints kinematics, starting from the estimation of the body segment orientation. Tong and Granat [7], calculated lower limb body segments orientation by integrating measured angular velocity from gyro sensors worn to the thigh and shank. However, integration caused errors to accumulate and caused the calculated values to ‘drift’ from the true value.

Many works in the past have reported methods to rectify this drift phenomenon. Takeda *et al.* [8] developed an optimization algorithm to extract only the gravitational acceleration component from the cyclic patterns in acceleration data during gait. Thus, this enabled the calculation of the inclination of lower body segments during gait without the usage of gyro sensors. Liu *et al.* [9] corrected the gyro drift by calculating the orientation of body segments from the acceleration during the mid-stance phase of gait. Favre *et al.* [10] used acceleration data to compensate for drift in the angular velocity data in situations where gravitational acceleration is the only component measured. Almost all the proposed methods have assumed that the measured gait contained cyclic patterns, however these method would not be applicable for subjects with impaired mobility, where cyclic properties of gait might not be apparent.

An alternate method, not using the cyclic patterns in gait, involved using two inertial sensors attached to the thigh and shank to estimate the acceleration sensor output at the center of the knee joint [11,12]. This method obtained accurate knee joint measurements and Takeda *et al.* [13] expanded a similar approach to estimate the positions of hip, knee and ankle joint in a three dimensional global coordinate system. Though the hip and knee joint flexion-extension motions showed high correlation with a reference camera system, the internal-external rotation motion was not considered.

Recently, three dimensional wearable sensors systems have been made commercially available, such as the MTx (Xsens Technologies B.V., Enschede, The Netherlands). These sensor systems combine geomagnetic sensors to correct the orientation deviation of angular velocity and acceleration data. This allowed the calculation of internal and external rotation. High reliability and accuracy was reported using these systems for gait analysis [14]. However, Brodie *et al.* [15] reported that three dimensional orientation accuracy errors existed, even during static states, when compared to a camera based analysis. These sensor systems required periodic recalibration and ferromagnetic materials in the environment affected measurement accuracy.

In this work gait analysis of five healthy volunteers was measured using a wearable sensor gait analysis system called H-Gait (Development Code, Laboratory of Biomechanical Design, Hokkaido University, Sapporo, Japan) [8,13,16]. The H-Gait system did not rely on an external magnetic field for reference and measurements were solely collected from orthogonally aligned tri-axial acceleration sensors and tri-axial gyro sensors which were fixed to seven locations on the lower limbs of each volunteer. The acceleration and angular velocity during level walking were collected. These measured data were then converted to three dimensional orientations of the wearable sensors, as well as the body segments they were attached to, using the H-Gait system's quaternion based algorithm developed by Tadano *et al.* [16].

The H-Gait system was developed and intended to be used in evaluating the walking ability and rehabilitation effects of patients with gait disorders. Normally in clinical practice 10 m walking tests are conducted for the evaluation of patients with gait irregularities related to stroke, spinal cord injuries (SCI), osteoarthritis (OA), multiple sclerosis (MS), *etc.* [17–19]. Unfortunately, very little has been reported where wearable sensors have been used in 10 m walking tests that provided both three dimensional kinematics and spatio-temporal gait parameters. Therefore the objective of this work was to implement the H-Gait system to the 10 m walking test and provide clinicians with kinematic and spatio-temporal gait parameters required for diagnosing a patient. However, before this some issues with the drift error caused by angular velocity integration had to be addressed. In order to reduce the error caused by signal drift, this work implemented several novel countermeasures. These novel methods include, a sensor attachment calibration protocol, designing a Butterworth filter, removing sensor offset values and a double derivative and integration method. By implementing these countermeasures the signal drift was significantly reduced. As a result, the proposed method could provide the lower limb joint kinematics such as the hip/knee/ankle flexion-extension (FE) angles, hip/knee adduction-abduction (AA) angles, and hip/knee/ankle internal-external (IE) rotation angles. Moreover, a moving wire frame model was created to visually confirm the gait motion. In addition, spatio-temporal parameters such as gait cycle (GC), cadence (CD), step length (SL), step width (SW), stride (STRL), limp index (LI), stand ratio (STR) and swing ratio (SWR) were calculated from the heel-contact (HC) and toe-off (TO) timing of the foot.

## 2. Method

The objective of this work was to develop a method based on wearable sensors that can accurately and quantitatively detect abnormalities during walking. A customized gait analysis set-up and measurements protocol, including the subject preparation, the initial acquisition and the following walking trials, were properly planned.

### 2.1. Drift Removal Countermeasures for Wearable Sensors

The gait posture can be calculated by finding the gravitational acceleration direction from the acceleration sensor and calculated the initial three dimensional orientation of the body segment to which the sensor was attached. The subsequent three dimensional orientations from the initial one can be estimated by integrating the angular velocity measured by the gyro sensors. However, one of the difficulties involved in this method is error caused by the accumulation of signal noise during this integration process. The integrated values will deviate from the true value, thus causing drift. The countermeasures taken to reduce the effect of signal drift will explained in the following.

### 2.2. Signal Noise Countermeasures

#### 2.2.1. Sensor Attachment Errors Reduction Protocol

**Figure 1.**
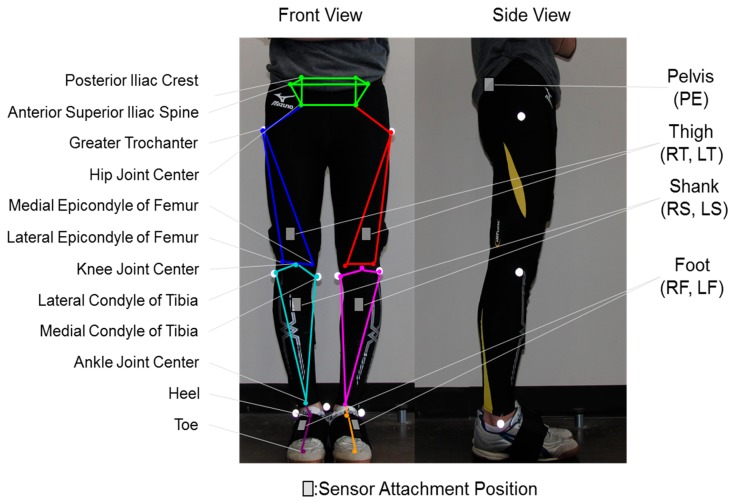
Sensor attachment location and gait wire frame model. The wire frame model is created by connecting characteristic positions of the lower limb. Sensor units are attached to seven body segments of the lower limb. This model has been implemented from the works of Tadano *et al.* [16].

A calibration procedure for decreasing the attachment errors of the sensors to the body segments was implemented. Figure 1 shows the three dimensional wire frame model implemented from the works of Tadano *et al.* [16]. The wire frame model is created using orientations of each body segment and the volunteer specific body measurements. The calibration method obtained the rotation matrix for converting the sensor to body segment coordinate system. This procedure involved two simple steps of measuring the gravitational acceleration vector for each lower limb segment in two different postures, standing and sitting. The wearable sensors were assumed to be aligned in a 2-D sagittal plane and a rotation matrix was derived to convert measurements of the sensor coordinate system to the global coordinate system. This implementation lead to minimizing the effects of attachment errors associated with wearable sensors.

#### 2.2.2. Digital Filtering Protocol

An infinite impulse response (IIR) digital 4th order Butterworth filter was implemented to remove noise from the raw gyro sensor data. This low pass filter was implemented using MATLAB (Mathworks, Natick, MA, USA), where the cutoff frequency was set to 12 Hz chosen according to the Nyquist theorem. The filter was applied in both the forward and backward direction to cancel out phase lag caused by the Butterworth filter.

In addition to this, offset values from the gyro sensor data were removed. This was done by obtaining the mode value of the gyro sensor data at a static state and subtracting it from the measured data for each individual axis of each sensor unit.

Concerning what was reported in previous works [16], the drift was not completely removed. Even for a measurement time of 14 s, drift appeared and influenced the final outcomes in terms of both joint kinematics and the wire frame model position and orientation in the space. While the previous method just attempted to remove the drift by subtracting from the entire signal the difference between the first and the last value of each angle of orientation of the sensor unit (roll, pitch and yaw), a more mathematically robust technique is here proposed, always assuming that the drift error linearly increases over time.

Based on this assumption, a double derivative and integration (DDI) method was implemented. Once the orientation angle *θ_out_i_(t)* of a sensor, along each *i* axis (x, y and z), was obtained, the true angle *θ_i_(t)* and the error *e_i_(t)* can be assumed as follows:

(1) $$\theta_{\text{out\_i}}(t)=\theta_{i}(t)+e_{i}(t)$$

After a double derivative operation, the quantity e*_i_(t)* can be removed. Assuming that the drift error linearly increasing over time, once derived it becomes a constant (*const*) that if derived again can be neglected, according to Equations (2) and (3):

(2) $$\frac{d(\theta_{\text{out\_i}}(t))}{dt}=\frac{d(\theta_{i}(t))}{dt}+\frac{d(e_{i}(t))}{dt}$$

(3) $$\frac{d^{2}(\theta_{\text{out\_i}}(t))}{dt^{2}}=\frac{d^{2}(\theta_{i}(t))}{dt^{2}}+\frac{d(\text{const})}{dt}$$

Considering that the orientation data is always necessary for the following analysis, a double integration was computed. This entails the addition of a constant of integration (c1 and c2), properly removed at each step of computation, as follows:

(4) $$\int {\ddot{\theta }}_{i}(t)dt={\dot{\theta }}_{i}(t)+c1$$

(5) $$\int {\dot{\theta }}_{i}(t)dt=\theta_{i}(t)+c2$$

The integration constant *c*1 was considered as the initial angular velocity. Since this study considered the start of any gait trial as a static state (stance phase), the initial angular velocity was 0. In addition, the integration constant *c*2 was considered as the initial orientation. Therefore the initial orientation calculated from the acceleration sensors at a static state (stance phase) was inputted into *c*2.

**Figure 2.**
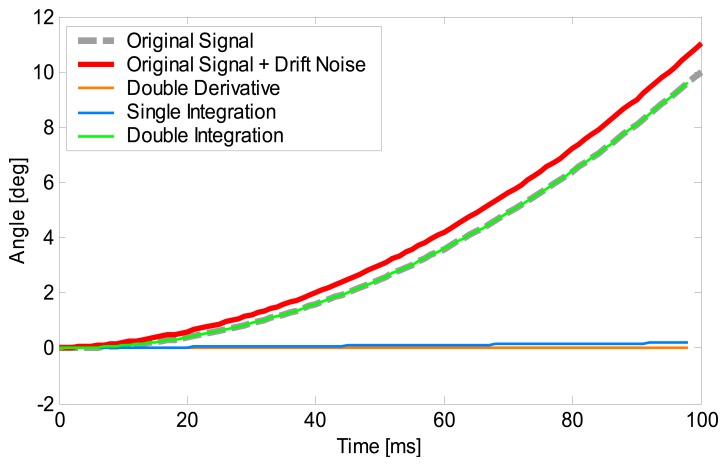
Simulation of the DDI method. The vertical axis represents the angle and horizontal axis represents time. The grey broken line represents the original signal and the red line represents the signal model with linear drift noise. The signal processed after double derivative, single integration and double integration are represented by the orange, blue and green lines, respectively.

Figure 2 shows the simulation of the process for Equations (2)–(5). The grey broken line represents the original signal (true value) and the red line represents the signal model with linear drift noise (original signal + drift noise). First, the original signal + drift noise is put through the double derivative process of Equations (2) and (3), the result is the orange line. Then the integration is implemented by Equation (4) and the initial angular velocity (in this case 0) is added as the integration constant (blue line). Finally, the double integration is completed by implementing Equation (5) and adding the initial orientation of the sensor as the integration constant (green line). The simulation shows that the signal after the DDI process (green line) coincides with the original signal (grey line). This is because the underlining assumption is that the drift included in the original signal increases linearly with time. Therefore the DDI method is able to eliminate the accumulation of drift. The drift error was removed according to both the signal processing used for noise and bias reduction of gyro sensors data and to the methodology aforementioned.

### 2.3. Gait Parameters: Spatio-Temporal Analysis and Joint Kinematics

Gait is usually defined in terms of temporal and spatial components, indicating the timing periods during which the gait events occur and both the position and orientation of limbs and joints in the space, respectively. A gait cycle is commonly divided into the stance and the swing phase; the first one starts with an initial foot contact, namely heel-contact (HC), while the second one starts with a toe-off (TO) event. Based on these main timing events, spatio-temporal gait parameters, such as GC, CD, SL, SW, STRL, LI, STR, SWR occurring during a gait cycle [1,20] can be derived.

In this study, the spatio-temporal parameters and gait phases were considered, as listed in Table 1, starting from the identification of both HC and TO timing events for each foot. It has been reported elsewhere that the HC and TO timings can be detected by accelerometers on the shank [21]. In this work these timing events were automatically identified directly from the angular velocity recorded from sensors placed on both shanks, with a MATLAB algorithm.

**Figure 3.**
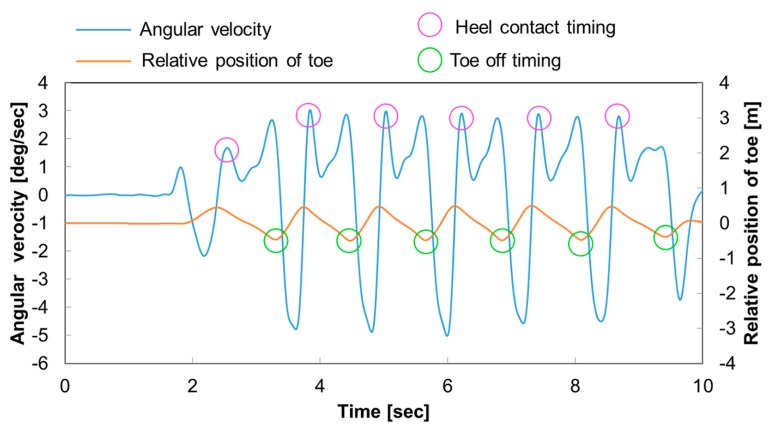
The method used to detect the HC and TO timing of the foot. The vertical axis represents the angular velocity and the relative position of the toe. The horizontal axis represents time. The HC timings are detected by the characteristic lateral angular velocity peaks and circled in pink. The TO timings are detected by measuring the negative peaks of the relative distance of the toe position to the origin of the pelvis (PE) coordinate system as circled in green.

**Figure 4.**
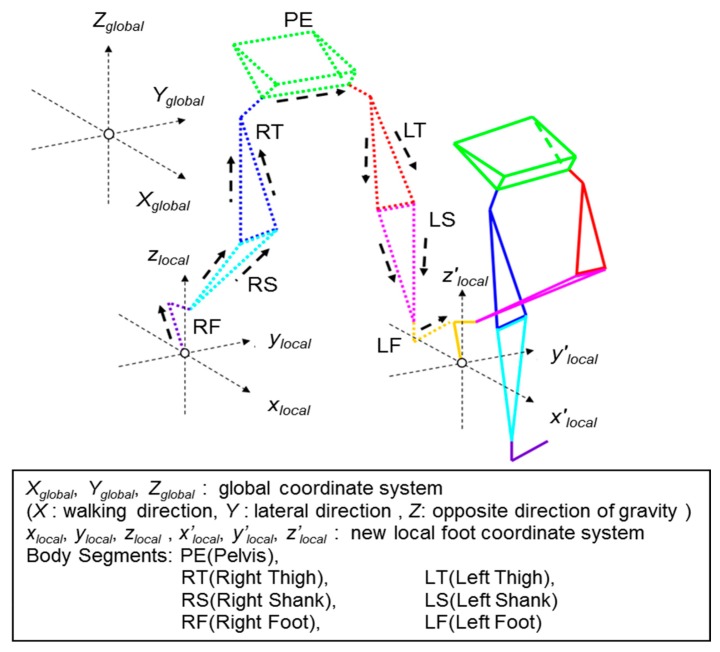
Wire frame model of the volunteer. The *X_global_*, *Y_global_*, *Z_global_* represent the global coordinate system, where the *X_global_* axis is the walking direction, the *Y_global_* axis is the left-lateral direction, and the *Z_global_* axis the vertical direction. The *x_local_*, *y_local_*, *z_local_* and *x′_local_*, *y′_local_*, *z′_local_* represent the new local foot coordinate system based on each step of gait. PE, RT, LT, RS, LS, RF and LF represent each of the body segments.

As shown in Figure 3, the HC are detected by the characteristic lateral angular velocity peaks and the TO timings are detected by measuring the negative peaks of the relative distance of the toe position to the origin of the pelvis (PE) coordinate system. Based on proper peaks of the angular velocity along the lateral axes of each segment, the gait cycles and stand ratio were calculated. The cadence, stride length, step length and step width were calculated by measuring the HC position of both legs. Figure 4 shows the relationship between the global coordinate system used in this work and the new local foot coordinate system created for each step. Each body segment is indicated as follows: pelvis (PE), right and left thigh (RT, LT), right and left shank (RS, LS), right and left foot (RF, LF). If the right foot (RF) is on the ground, during the HC to foot flat (FF), local foot coordinate system *x_local_*, *y_local_*, and *z_local_* are created from the heel position of RF. From FF until the HC of left foot (LF), local foot coordinate system *x_local_*, *y_local_*, and *z_local_* are created from the toe position of RF. The other body segment three dimensional orientations in the global coordinate system are calculated based on their relative orientation against RF. Therefore, the body segment three dimensional orientations in the global coordinate system will be calculated in the order of RF→RS→RT→PE→LT→LS→LF. This order will continue until LF is on the ground where a new local foot coordinate system *x′_local_*, *y′_local_*, and *z′_local_* is created and the orientation calculation order will start from LF→LS→LT→PE→RT→RS→RF.

Joint kinematics in terms of gait trends are commonly analyzed during a clinical gait analysis session. In this work, joint kinematic gait parameters such as the hip/knee/ankle flexion-extension (FE) angles, hip/knee adduction-abduction (AA) angles, and hip/knee/ankle internal-external (IE) rotation angles can be calculated.

## 3. Experiments

Experiments were conducted indoors on a straight flat floor with five healthy volunteers. The volunteers' body measurements were taken before experiment and are shown in Table 2. Body measurements such as the distance of the greater trochanter (GT) to the lateral condyle of tibia (LCT), LCT to ankle joint, ankle joint height and the right GT to left GT width. For calculation simplification purposes the body measurements of the healthy subjects were assumed to be bilaterally symmetric and only the body measurements of the right limb were measured.

The volunteers' were asked to do one gait trial consisting of standing still, 10 m walking test and then to standing still again. The volunteers wore spandex tracksuit pants with seven small pockets, for each body segment, fitted to hold the H-Gait system's wearable sensor units. Reflective markers were placed on 10 anatomical characteristic positions of the lower limb and a still picture was taken from the front and the two sides (Figure 1). The walking distance was approximately 10 m, equivalent to 10 steps (5 steps for each right and left leg).

## 4. Results

**Figure 5.**
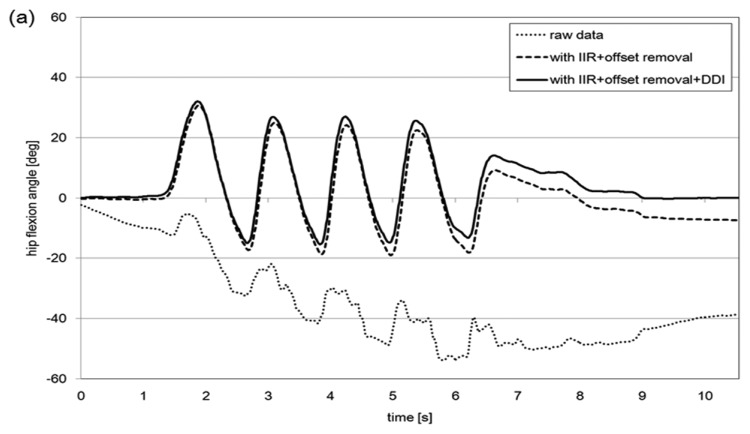

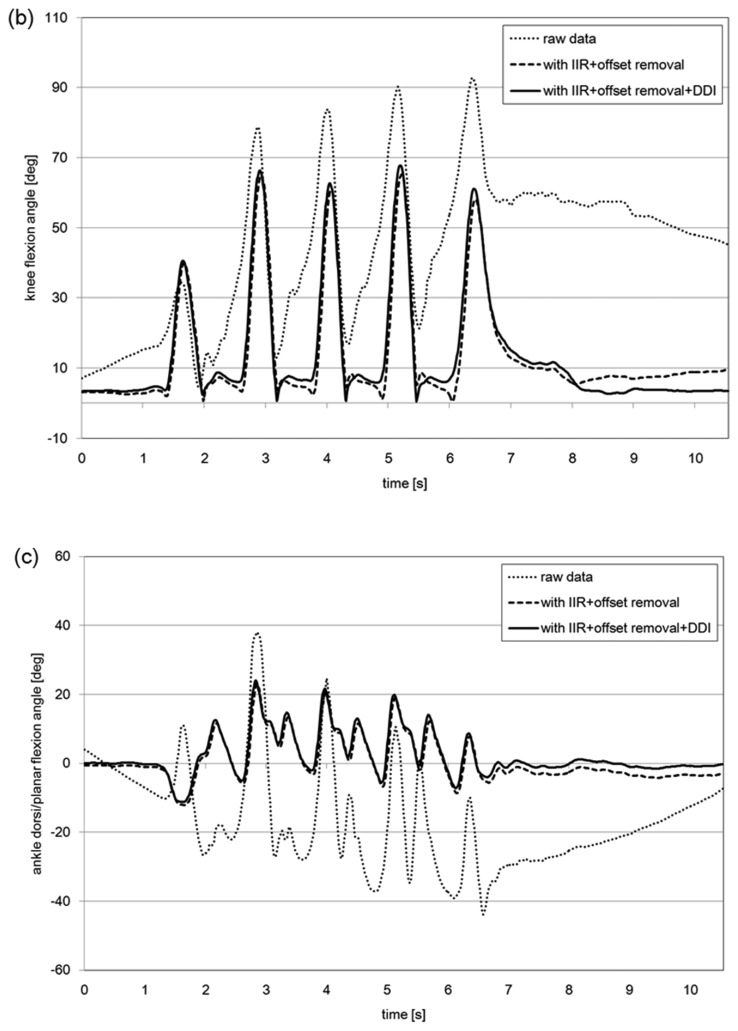
Comparison of the joint angle results obtained through the different methods of signal drift reduction protocol (raw data, IIR + offset removal, IIR + offset removal + DDI) for a volunteer. (**a**) Hip joint flexion angle; (**b**) knee joint flexion angle and (**c**) ankle joint flexion angle are shown.

The comparison of the joint angle results obtained through the different methods of signal drift reduction protocol (raw data, IIR + offset removal, IIR + offset removal + DDI), for each lower limb joint (hip joint flexion angle, knee joint flexion angle and ankle joint flexion angle) are shown in Figure 5.

The results are an example of a volunteer over a period of 10 s of walking. The raw data, represented by the dotted line, is the joint angle calculated without implementing any of the signal drift protocols, thus the effect of error is large. During about 1.4–7.8 s, while the volunteer was performing the gait motion, the drift increased linearly. This is the reason why DDI method was a plausible method to remove this linear drift effect during dynamic states such as gait. The IIR + offset removal protocol, represented by the broken line, refers to the method proposed by Tadano *et al.* [16]. The IIR + offset removal + DDI protocol, represented by the solid line, represents the results from this work.

**Figure 6.**
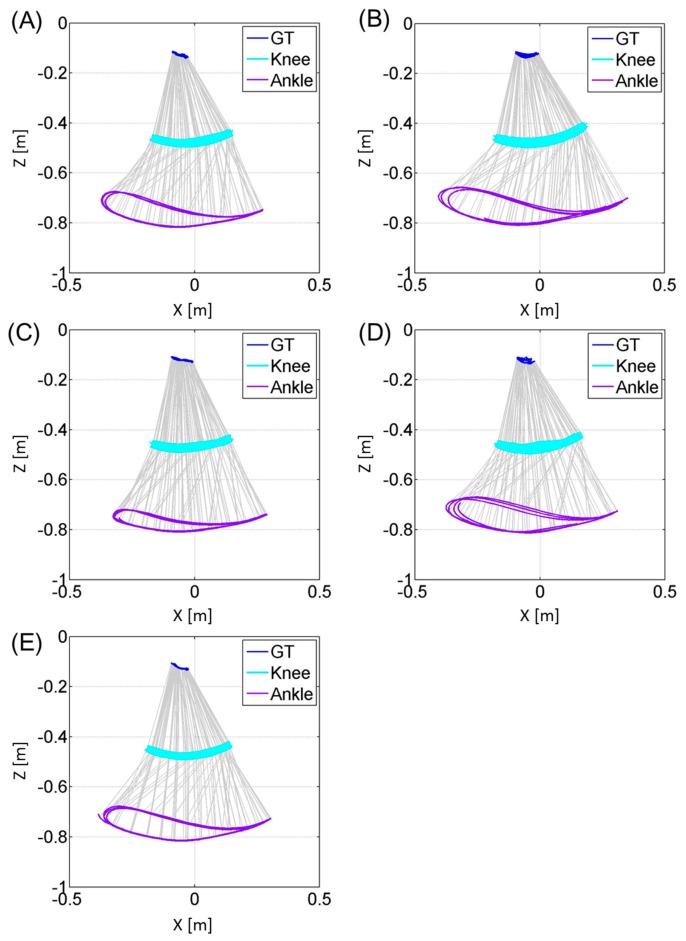
These figures represent the trajectories of the greater trochanter (GT), knee joint center and ankle joint center for the right leg of each subject (**A**, **B**, **C**, **D**, **E**) during three gait cycle in the sagittal plane. The vertical axis represents the *Z_global_* axis and the horizontal axis represents the *X_global_* axis. The trajectories are plotted at a sampling rate of 33 Hz.

**Figure 7.**
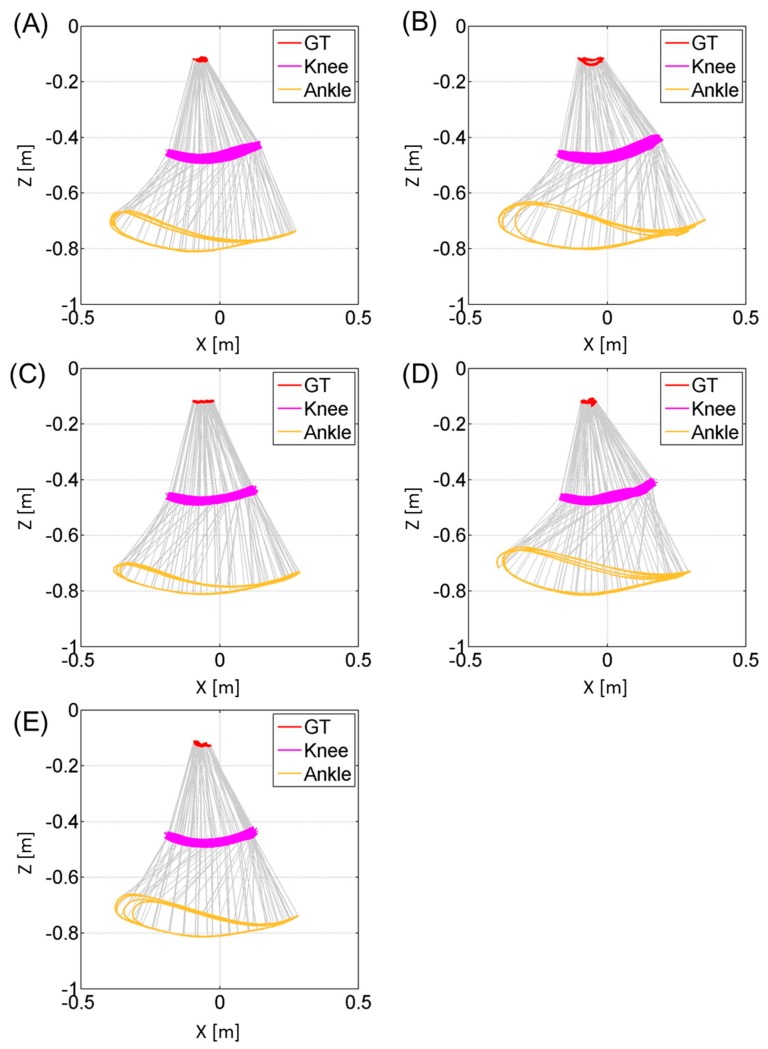
These figures represent the trajectories of the greater trochanter (GT), knee joint center and ankle joint center for the left leg of each subject (**A**, **B**, **C**, **D**, **E**) during three gait cycles in the sagittal plane. The vertical axis represents the *Z_global_* axis and the horizontal axis represents the *X_global_* axis. The trajectories are plotted at a sampling rate of 33 Hz.

**Figure 8.**
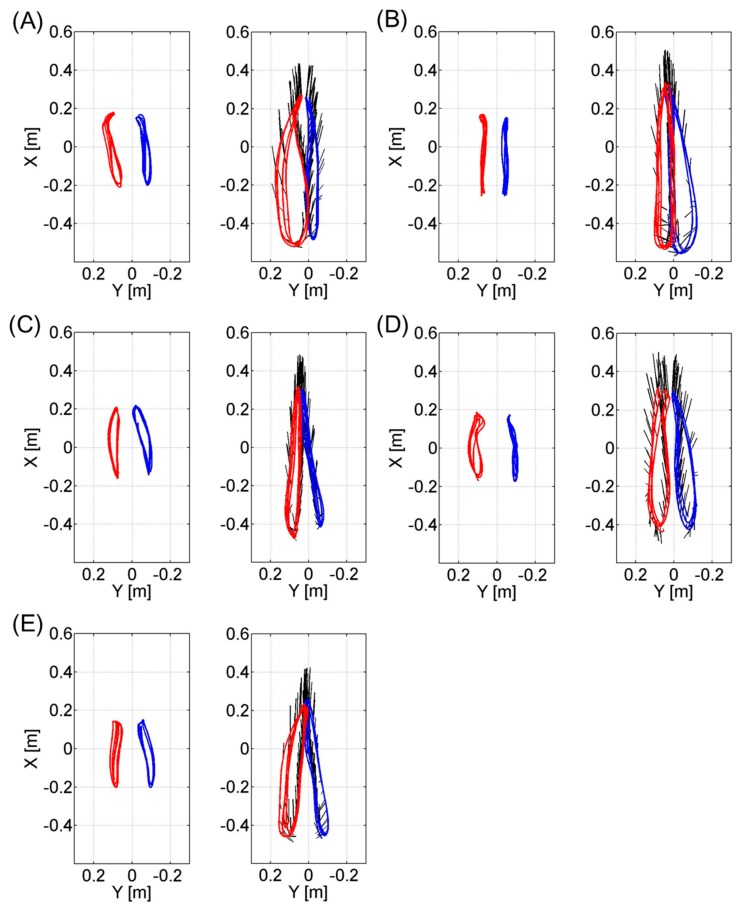
These figures represent the trajectory of the knee (**left** figure) and ankle joint (**right** figure) center during three gait cycles in the horizontal plane for each volunteer sampled at 33 Hz. The vertical axis represents the *X_global_* axis and the horizontal axis represents the *Y_global_* axis. The trajectories for the left knee/ankle are shown in red and those for the right knee/ankle are in blue. The black line emanating from the ankle joint trajectories represent the direction of each toe. (**A**) through (**E**) represent the data for each of the five volunteers.

Figures 6 and 7 represent the trajectories of the right GT and left GT, knee joint center and ankle joint center in the sagittal *Z_global_*-*X_global_* plane respectively. Both Figures 6 and 7 are plotted at a sampling rate of 33 Hz. The trajectories for the knee joint center and ankle joint center trajectories projected on the horizontal *X_global_*-*Y_global_* plane are shown in Figure 8. Both sagittal and horizontal trajectories results are that of three gait cycles of each subject plotted at 33 Hz. Table 3 shows the result of the spatio-temporal parameters for each subject. Here the gait cycle, cadence, step length, step width, stride length, limp index, stand ratio and swing ratio are shown.

## 5. Discussion and Conclusions

The objective of this work was to propose a method for removing the effect of drift for improving the measurement accuracy of gait using wearable sensors. The results show that implementation of a combination of countermeasures; sensor attachment errors reduction protocol, IIR digital 4th order Butterworth filter, offset removal and the DDI method, were successful in reducing the effect of signal drift.

The results from Figure 5 show differences in the joint angles after about 10 s of gait. The average difference for all 5 volunteers, after 10 s, were 2.1°, 33.3° and 15.6° for the hip, knee and ankle joint angles, respectively, when comparing between RAW and IIR + offset removal + DDI. In addition, the difference between implementing IIR + offset removal and IIR + offset removal + DDI was 6.2°, 6.6° and 2.2° for the hip, knee and ankle joint angles, respectively. This shows that the proposed countermeasure allowed for an average of 17° of drift error removal compared to integrating raw angular velocity data and an average of 5° less drift error than previous reported methods [16].

The results of Figures 6 and 7 show that this method is able to visually compare the differences in the joint trajectories between the right GT and left GT, knee and ankle joint centers using the wire frame model in the sagittal plane. These kinematic gait parameters will allow the comparison of the flexion-extension angles of the knee during different timings of the gait cycle. Figure 8 allowed for the comparison of the knee and ankle joint center relative trajectories in the horizontal plane. This data can compare the bilateral symmetry of both right and left knee joint and ankle joint trajectories. In addition the spatio-temporal parameters provided in Table 3 allow us to quantify the differences in the gait events between the right and left side. By combining both the kinematic and statio-temporal parameter, we can detect differences in gait of the left and right lower limb and see how the differences affect the orientation of body segments and ultimately the joint positions during gait.

The method reported here proved the feasibility of three dimensional gait analysis with the wearable sensor gait analysis system H-Gait in detecting gait events providing both kinematic and spatio-temporal parameters. However, the methodology introduced here is not exclusive to the H-Gait system and can be applied to other commercial wearable sensors systems using acceleration and gyro sensors. These data will be useful in clinical sites, such as those for the follow-up diagnosis after TKA, by using the gait parameters in order to quantify the recovery of walking [22,23] or on the influence of a specific implants designs on gait [24].

A limitation of this work lies in the sensor attachment error calibration procedure. This procedure assumed that the orientation change in the stand and sitting posture of the wearable sensors changed in the 2-D sagittal plane only. However if other motions, such as internal-external or varus-valgus rotations of the knee, were to take place in between the two postures, this would cause error in the results. Therefore fixing the knee joint more securely to insure that the internal-external and varus-valgus rotation does not interfere with the calibration maybe required. Another method would be to introduce another posture other than stand and sitting to consider the 3-D attachment error. Both possibilities will be investigated in future works.

The methodology reported here has implemented a mathematical technique for the drift reduction, in addition to the previous work [16]. It was always assumed that drift linearly increased over time, taking into account that measurements were limited to about 10 s of gait. Elsewhere it has been reported that for long measurement times such as over 100 s the drift effect may not linearly increase [25,26]. Therefore, for longer distances applications, a more robust method like the Kalman filters or empirical mode decomposition (EMD) techniques may have to be considered to effectively remove drift.

Recently, fusing tri-axial gyroscope and tri-axial acceleration data from an IMU via an optimized Kalman filter was proposed by Mazza *et al.* [27]. The RMSE between the proposed method and an optical tracking system were less than 1.0°. Yuan and Chen developed a method, using a combination of an acceleration tuning algorithm and a Kalman filter, to remove drift from 3 IMUs [28]. They reported accuracy within 2% of the total walking length. However, one of the draw backs of designing a complicated Kalman filter is the requirement of fine tuning or optimizing the internal filter parameters to gain accurate estimates. In addition, these parameters will vary according to the kind of IMU used and the signal noise of the environment. Therefore, the specification of the IMU, the surrounding noise level and the target motion must be understood to before designing a good Kalman filter. Bonnet *et al.*, introduced a method not requiring tuning by implementing an EMD technique [29]. Though the result RMSE between the proposed method and an optical tracking system was less than 2.1°, it was more robust than using a Kalman filter.

The work presented here has not been compared against a reference optical motion tracking system. A comparison would have been useful to provide a more detailed confirmation surrounding the effectiveness of the proposed method. However, the comparison with an optical tracking system has already been discussed in previous studies [16] and even without a detailed accuracy comparison, it has been reported that simple two dimensional IMU measurements were capable of providing kinematic and spatio-temporal gait parameters for comparing the paretic and non-paretic leg of a stroke patient [30].

In conclusion, it can be said the methodology required to remove the drift effect depends on the wearable sensors used, the kind and distance of the gait measured. As stated in the introduction, this work intended the use of wearable sensors in measuring the normal 10 m walking test of patients. In most cases these patients have a hard time of even walking 10 m and asking them to walk even more is not practical. Therefore with the target walking distance limited to about 10 m, the proposed method here sufficiently reduces the effect of drift and allows a fairly accurate gait analysis of patients.

Following this study, other gait phases and parameters should be calculated and investigated more in the future, in order to analyze e.g., also other gait events, like the foot-flat phase in addition to the heel-contact and toe-off ones. Concerning the methodology used to manage the drift problem, a nonlinear assumption could be considered for future works.

## Figures and Tables

**Table 1.** The spatio-temporal gait parameters defined in this work.

**Parameter**	**Definition**
Gait cycle [s]	Duration of a gait cycle
Cadence [step/min]	Number of steps/min
Step length [cm]	Distance between a heel-strike (or toe-off) of both feet
Step width [cm]	Distance between medial lines of feet
Stride [cm]	Distance between two heel-strikes (or toe-offs) of one foot
Limp Index	Ratio of stance of subject limb *vs.* opposite limb
Stand ratio [%]	Period of a gait cycle in which the foot is on the ground.
Swing ratio [%]	Period of a gait cycle in which the foot is off the ground.

**Table 2.** Information of the volunteers A to E. Body segment measurements between each characteristic anatomical point are used. The lengths are those measured from the right limb only.

		**GT to LCT [cm]**	**LCT to Ankle [cm]**	**Ankle Height [cm]**	**Right GT to Left GT Width [cm]**

Subject	A	40.8	43.0	8.9	35.0
	B	39.4	41.5	9.5	34.3
	C	42.5	42.7	8.5	35.8
	D	36.0	37.8	11.5	34.0
	E	37.0	39.0	11.0	36.0

**Table 3.** Spatio-temporal gait parameters for each subject (A, B, C, D, E) for both right and left lower limb. Gait cycle, cadence, step length, step width, stride length, limp index, stand ratio and swing ratio are shown with mean and standard deviation.

			**A**	**B**	**C**	**D**	**E**	**Mean**	**SD**

Global		Gait cycle [sec]	1.20	1.18	1.27	1.16	1.18	1.20	0.04
		Cadence [step/min]	99.79	101.76	94.30	103.52	102.06	100.29	2.94
		Step length [cm]	76.17	80.39	76.19	74.44	69.56	75.35	3.19
		Step width [cm]	15.73	16.36	21.56	19.63	20.45	18.75	2.10
		Stride [cm]	151.58	155.18	149.80	148.60	139.62	148.96	4.72

Differentiated	Right	Limp index	0.98	0.88	1.08	0.97	1.00	0.98	0.06
		Stand ratio [%]	39.66	42.62	31.27	40.11	47.37	40.21	4.78
		Swing ratio [%]	60.34	57.38	68.73	59.89	52.63	59.79	4.78

	Left	Limp index	1.02	1.14	0.92	1.03	1.00	1.02	0.06
		Stand ratio [%]	40.15	41.91	36.69	42.43	43.37	40.91	2.15
		Swing ratio [%]	59.85	58.09	63.31	57.57	56.63	59.09	2.15
